# The Use of Nutritives and Tonics in Nervous Disease

**Published:** 1890-05

**Authors:** 


### THE USE OF NUTRITIVES AND TONICS IN NERVOUS DISEASE.

In all cases of vital depression it is a matter of the first importance, that the physical system be reinforced by nutritives and tonics.

This vital depression is nearly always a feature, and generally a marked one, of nervous and mental diseases; of "brain fag" and "nervous prostration," and under no other circumstances are nutritives and tonics more clearly demanded or potent for good. There is much choice in remedies of this class. Dr. Wm. Stocker, physician at Mt. Hope Retreat, near Baltimore, clearly indicates his preference:

"I have for some time past been in the habit of using "Colden's Liquid Beef Tonic" in my private practice, and have also tested its qualities in cases under my care at Mt. Hope Retreat. I have found it an admirable nutritive tonic and stimulant, and in a great variety of cases accompanied by serious vital depression, and in tedious convalescence from want of appetite, and inability to digest the more common articles of aliment, "Colden's Liquid Beef Tonic" will be found the very best preparation used, and I, therefore, confidently recommend it to the medical profession."—*Mass. Med. Journal.*

---

The report of the New York Analyst of Drugs shows that the chances for getting drugs of good quality on prescription is 43.8 per cent.; fair, 17.4; inferior, 26.; NOT AS CALLED FOR. 11.6; excessive strength, 1.2—(*Times and Register.*

Philadelphia, December 7, 1889.

